# Antimicrobial-resistant infections among postpartum women at a Ugandan referral hospital

**DOI:** 10.1371/journal.pone.0175456

**Published:** 2017-04-13

**Authors:** Lisa M. Bebell, Joseph Ngonzi, Joel Bazira, Yarine Fajardo, Adeline A. Boatin, Mark J. Siedner, Ingrid V. Bassett, Dan Nyehangane, Deborah Nanjebe, Yves Jacquemyn, Jean-Pierre van Geertruyden, Juliet Mwanga-Amumpaire, David R. Bangsberg, Laura E. Riley, Yap Boum

**Affiliations:** 1 Division of Infectious Diseases, Massachusetts General Hospital, Boston, MA, United States of America; 2 Massachusetts General Hospital Center for Global Health, Boston, MA, United States of America; 3 Mbarara University of Science and Technology, Mbarara, Uganda; 4 Global Health Institute, University of Antwerp, Antwerp, Belgium; 5 Division of Obstetrics and Gynecology, Massachusetts General Hospital, Boston, MA, United States of America; 6 Epicentre Mbarara Research Centre, Mbarara, Uganda; Centers for Disease Control and Prevention, UNITED STATES

## Abstract

**Introduction:**

Puerperal sepsis causes 10% of maternal deaths in Africa, but prospective studies on incidence, microbiology and antimicrobial resistance are lacking.

**Methods:**

We performed a prospective cohort study of 4,231 Ugandan women presenting to a regional referral hospital for delivery or postpartum care, measured vital signs after delivery, performed structured physical exam, symptom questionnaire, and microbiologic evaluation of febrile and hypothermic women. Malaria rapid diagnostic testing, blood and urine cultures were performed aseptically and processed at Epicentre Mbarara Research Centre. Antimicrobial susceptibility and breakpoints were determined using disk diffusion per EUCAST standards. Hospital diagnoses, treatments and outcomes were abstracted from patient charts.

**Results:**

Mean age was 25 years, 12% were HIV-infected, and 50% had cesarean deliveries. Approximately 5% (205/4176) with ≥1 temperature measurement recorded developed postpartum fever or hypothermia; blood and urine samples were collected from 174 (85%), and 17 others were evaluated clinically. Eighty-four (48%) had at least one confirmed source of infection: 39% (76/193) clinical postpartum endometritis, 14% (25/174) urinary tract infection (UTI), 3% (5/174) bloodstream infection. Another 3% (5/174) had malaria. Overall, 30/174 (17%) had positive blood or urine cultures, and *Acinetobacter* species were the most common bacteria isolated. Of 25 Gram-negatives isolated, 20 (80%) were multidrug-resistant and cefepime non-susceptible.

**Conclusions:**

For women in rural Uganda with postpartum fever, we found a high rate of antibiotic resistance among cultured urinary and bloodstream infections, including cephalosporin-resistant *Acinetobacter* species. Increasing availability of microbiology testing to inform appropriate antibiotic use, development of antimicrobial stewardship programs, and strengthening infection control practices should be high priorities.

## Introduction

The World Health Organization (WHO) estimates puerperal sepsis causes 10% of maternal deaths in Africa.[1] However, prospective studies of pregnancy-related infections are scant, and most data come from retrospective studies of maternal deaths without formal microbiological investigation or antimicrobial susceptibility testing.[2–6] Postpartum infections are pregnancy-related infections occurring between the onset of rupture of membranes in labor and the 42^nd^ day after delivery, and are important causes of morbidity and mortality worldwide. The most common postpartum infections include endometritis (puerperal sepsis), urinary tract infections (UTIs), bloodstream infections, and cesarean surgical site infections.[3, 5, 7] Cesarean delivery is considered the single most important risk factor for postpartum infection due to skin and uterine disruption, bladder catheterization, and healthcare system contact, though peri-procedural antibiotic prophylaxis reduces the risk significantly.[3, 8]

To date, most research on postpartum infection has occurred in high-resource settings. In low-resource settings, differences in antibiotic prophylaxis and treatment, cesarean delivery practices, human immunodeficiency virus (HIV) prevalence, access to microbiology testing, antimicrobial stewardship and infection control practices may lead to differences in infection incidence, microbiology, and antimicrobial resistance.[4, 5, 9, 10] To address this gap in knowledge, we performed a prospective cohort study of women presenting to a Ugandan regional referral hospital for delivery or care within the 42-day postpartum period. We sought to better define the incidence, microbiology, and antimicrobial resistance of postpartum infections in this resource-limited setting to guide antibiotic selection, urinary catheter use, and antimicrobial stewardship.

## Methods

### Study site

Participants were recruited from Mbarara Regional Referral Hospital (MRRH) in Mbarara, Uganda between March and October, 2015. MRRH is an approximately 300-bed academic hospital affiliated with Mbarara University of Science and Technology (MUST). It is the largest teaching and referral hospital for Western Uganda, with population of nine million people living in a predominantly rural, agrarian setting. According to local standard of care, women hospitalized for delivery and postpartum care undergo daily and as-needed vital signs monitoring. Vaginal deliveries are attended by midwives, while cesarean deliveries are conducted by obstetricians. Current hospital policy is for women delivering by cesarean to receive a single dose of peri-procedural antibiotic prophylaxis (ampicillin or ceftriaxone), usually given within 30 minutes of skin incision. In addition, after cesarean delivery women are treated with combination intravenous ceftriaxone and metronidazole for three days, followed by five days of oral cefixime. Antibiotics are not routinely given to women delivering vaginally. Microbiological evaluation of potential infections is not routine, but when requested, samples are processed at MUST’s teaching lab adjacent to MRRH. There, bacterial identification is performed using traditional biochemical methods with limited supplies.

### Participant recruitment and clinical data collection

All women presenting to MRRH maternity ward in labor for delivery or for care within 42 days postpartum were approached for enrollment, including women hospitalized for antenatal care who subsequently labored. All study participants provided written informed consent. Potential participants were excluded if they did not speak English or Runyankole, if they declined consent, or were incapacitated and next-of-kin declined surrogate consent. Enrolled women were followed by a team of trained research nurses measuring vital signs including oral temperature approximately every eight hours after delivery for the duration of the participant’s hospitalization.

### Sample collection, microbiology and antimicrobial susceptibility testing

Participants with fever >38.0°C or hypothermia < 36.0°C were tested for malaria using SD Bioline Malaria Ag Pf/Pan rapid diagnostic test (Standard Diagnostics, Gyeonggi, Korea), provided a clean-catch urine sample, and had peripheral blood drawn aseptically into Becton Dickinson (BD) BACTEC (Becton, Dickinson and Company, Franklin Lakes, USA) bottles. Two aerobic, two anaerobic and one mycobacterial blood culture were transported to the Epicentre Mbarara Research Centre microbiology laboratory adjacent to MRRH. Epicentre complies with Good Clinical Laboratory Practice standards, is seeking accreditation with Strengthening Laboratory Management Toward Accreditation (SLMTA), and is overseen by Epicentre France, whose staff perform regular on-site quality control and training. On arrival to Epicentre, blood culture bottles were incubated in a BD BACTEC 9240 automated blood culture system for seven days before reporting no growth. Mycobacterial cultures were reported negative after 42 days’ incubation. When the BACTEC system detected growth, Gram stain and sub-culture were performed, using enriched and selective media for subculture. Urine samples were obtained after participants self-cleansed the perineum with castile soap towlettes (for non-catheterized participants) or through nurse-collected aspirate of 10mL urine from externally-cleansed catheter tubing (for catheterized participants). Urine was transported to Epicentre in sterile collection containers and refrigerated at 2–8°C for not more than 12 hours. Urinalysis was performed using Wellkang (London, England) commercial test strips. After macroscopic and microscopic examination, urine was incubated on CHROMagar Orientation medium (CHROMagar, Paris, France) and incubated at 37°C until growth was observed or three days, whichever came first. If three or fewer colony types were isolated, Gram stain, subculture, colony counts, and bacterial identification were performed. Bacterial colonies were identified using standard biochemical methods and confirmed using the Analytical Profile Index system (bioMérieux, Marcy-l’Étoile, France) and antimicrobial susceptibility was determined using Kirby-Bauer disk diffusion. Antibiotic breakpoints were defined using European Committee on Antimicrobial Susceptibility Testing (EUCAST) guidelines, version 5.0.[11] *Enterobacteriaceae* testing intermediate or resistant to 3^rd^ or 4^th^ generation cephalosporins were evaluated for extended-spectrum β-lactamase (ESBL) production and considered ESBL phenotype if synergy was observed between amoxicillin/clavulanic acid and ceftazidime or cefotaxime. ESBL testing was not performed for non-lactose-fermenting Gram-negative rods.

### Participant demographic, medical, treatment, and outcomes data

Febrile or hypothermic participants and a random selection of normothermic participants underwent additional in-depth data collection including interview and chart review. We randomly selected 1,581 normothermic participants as a comparison group to achieve at least a 4:1 ratio of normothermic:febrile/hypothermic participants, maximizing power to detect differences between groups. Demographic characteristics and health conditions were obtained using a structured face-to-face interview in English or Runyankole created by study investigators. Medical charts were reviewed at hospital discharge to determine outcomes, diagnoses and treatments received. Though antibiotic prescription is well-documented in charts, antibiotic administration is rarely recorded.

### Defining infectious outcomes

Febrile and hypothermic participants were evaluated at the time of abnormal temperature using structured physical exam and symptom questionnaire developed by study investigators and administered by trained study nurses. Postpartum endometritis (puerperal sepsis) was defined using the WHO technical working group 1992 definition as infection of the genital tract in which two or more of the following were present: pelvic pain, fever >38.0°C, abnormal vaginal discharge, and delay in the rate of reduction of the size of the uterus <2cm/day.[12] Uncomplicated UTI was defined by urinalysis positive for leukocyte esterase or nitrite and urine culture growing ≥10^5^ colony-forming units (CFU) per milliliter (mL) of one or two pathogens in the absence of nausea, vomiting, urinary catheterization, or flank pain.[13] Pyelonephritis was defined by urine culture growing ≥10^3^ CFU/mL of one or two pathogens in the presence of nausea, vomiting or flank pain.[13] Catheter-associated urinary tract infection (CAUTI) was defined by urine culture growing ≥10^3^ CFU/mL of one or two pathogens with a urinary catheter in place or removed within the previous 48 hours.[14] Bloodstream infection (bacteremia) was defined as the growth of a potential pathogen in one or more blood culture bottles. Diagnosis of superficial cesarean surgical site infection of the abdominal wall or skin was abstracted from chart review. Our primary study outcome was confirmed postpartum infection, a composite outcome including bloodstream infection, urinary tract infection, or clinical endometritis diagnosed by our research staff using the definitions above. Cesarean surgical site infection was not confirmed by research staff and not included in the composite primary outcome.

### Data entry and statistical analysis

Questionnaires and laboratory results were entered into a Research Electronic Data Capture (REDCap) database.[15] Demographic characteristics and outcomes were compared between febrile/hypothermic participants and normothermic participants using Chi^2^ analysis. Additional analyses compared febrile/hypothermic women with a laboratory or clinically-confirmed source of infection to those without a confirmed source. *P*-values<0.05 were considered statistically significant. All analyses were performed using Stata software (Version 12.0, StataCorp, College Station, TX).

### Ethics

All enrolled women provided written informed consent prior to data collection. Participants <18 years of age were enrolled as emancipated minors based on pregnancy status. The study and enrollment procedures were approved by the institutional ethics review boards at Mbarara University of Science and Technology (08/10-14), Partners Healthcare (2014P002725/MGH) and the Uganda National Council of Science and Technology (HS/1729).

## Results

### Enrollment and demographics

**Fig 1:**
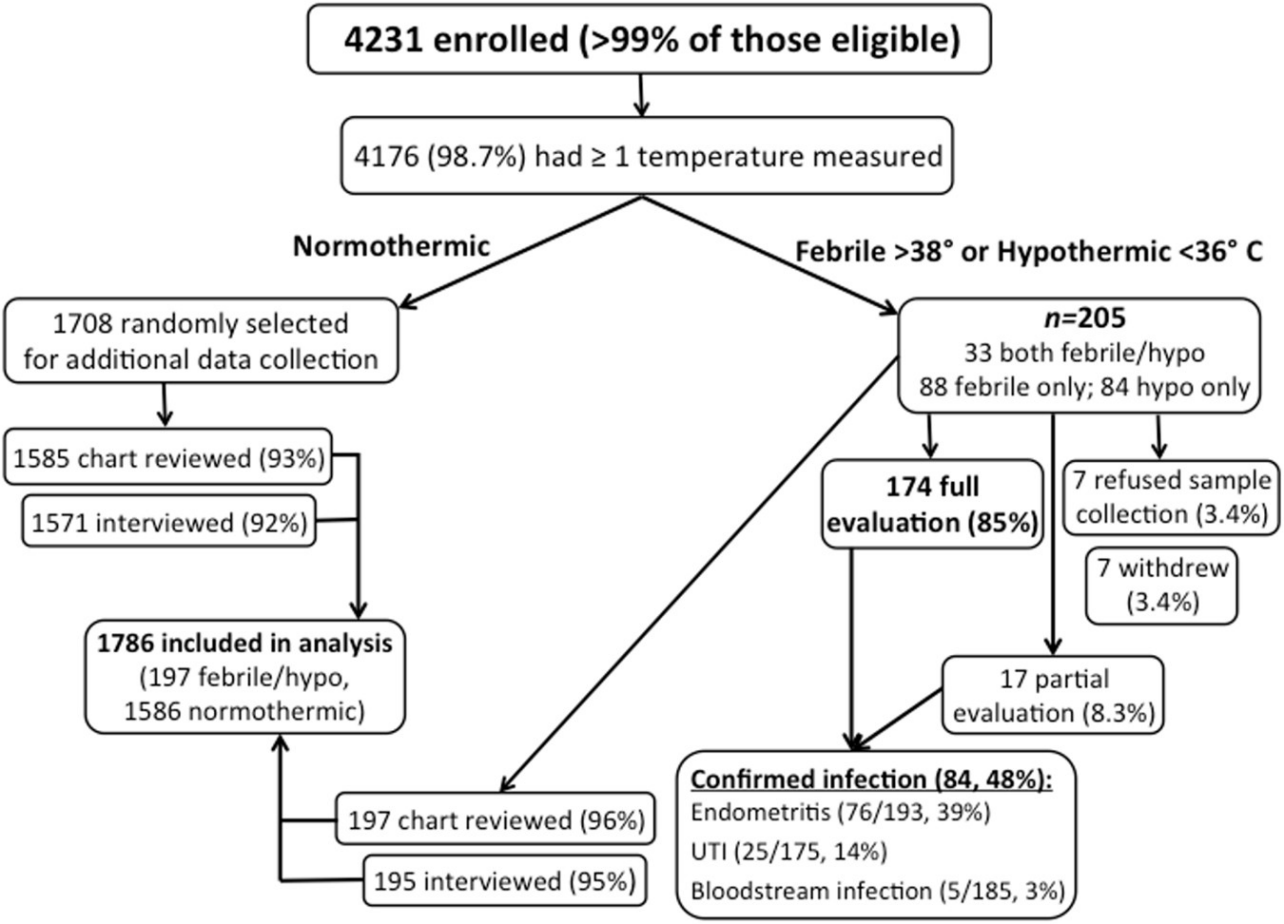
Enrollment flowchart demonstrating participants enrolled, retained and evaluated at each stage as well as confirmed infectious diagnoses.

Of all eligible women presenting to MRRH for care during the study period, over 99% (4,235) were enrolled, and four withdrew before data collection was performed. Of the normothermic cohort, 1,786 women were randomly selected for additional in-depth data collection. Mean cohort age was 25.2 years (standard deviation (SD) 5.5 years), 667 (38%) were primiparous, 875 (50%) delivered by cesarean, and 212 (12%) were HIV-1 infected (Table 1). Overall, 1,567 (92%) had both chart review and interview data, and 1586 with either chart review or interview data were included in the analysis (Fig 1).

**Table 1:** Demographic characteristics of a cohort of women presenting to a Ugandan referral hospital for delivery or postpartum care, comparing febrile or hypothermic women with microbiologically-confirmed infection to febrile or hypothermic women in whom no infectious source was found.

	Total	No confirmed	Confirmed	
	cohort	infection	infection	
Characteristic	(N = 1786)	(*n* = 90)	(*n* = 84)	*P*-value*
Age (years, mean, SD^a^)	25.2 (5.5)	24.4 (5.3)	22.6 (4.9)	0.02
Age category				0.07
<18	41 (2)	2 (2)	4 (5)	
18–24	849 (49)	46 (53)	57 (68)	
25–34	724 (41)	34 (39)	22 (26)	
>34	133 (8)	5 (6)	1 (1)	
Married (n, %)	1642 (93)	79 (91)	77 (92)	0.84
Residence in Mbarara (n, %)	728 (43)	23 (30)	27 (34)	0.60
Antenatal attendance ≥ 4 times	1249 (71)	57 (66)	65 (77)	0.09
Formally employed outside the home	684 (39)	21 (24)	23 (27)	0.63
Primiparous	667 (38)	36 (41)	57 (68)	0.001
Single gestation	1706 (97)	85 (97)	79 (94)	0.43
>5 vaginal exams during labor	180 (11)	9 (11)	6 (8)	0.53
Delivery mode				0.20
Spontaneous vaginal	855 (49)	18 (21)	10 (12)	
Breech or assisted vaginal	8 (0.5)	0 (0)	1 (1)	
Cesarean	875 (50)	68 (79)	71 (86)	
HIV^b^ infected	212 (12)	8 (9)	12 (14)	0.27
CD4^c^ category				0.75
< 250	64 (14)	0 (0)	2 (18)	
250–349	58 (13)	1 (13)	2 (18)	
350–500	108 (24)	2 (25)	2 (18)	
> 500	213 (48)	5 (63)	5 (46)	
Admitted in labor	1571 (90)	73 (85)	73 (89)	0.43
Days postpartum at admission (if not in labor; mean, SD^a^)	12.9 (19.2)	7 (5.7)	5 (4.6)	0.72
Received peri-Cesarean antibiotic prophylaxis	802 (92)	59 (98)	63 (100)	0.20
Urinary catheter placement	918 (52)	69 (79)	75 (89)	0.07
Urinary catheter duration (days, mean, SD^a^)	2.0 (1.1)	2.2 (1.2)	2.4 (1.5)	0.36

Table 1 footnote

^a^SD: standard deviation

^b^HIV: human immunodeficiency virus

^c^CD4: CD4+ T-cell count.

*P-values were obtained using Chi squared statistic, or t-test for continuous normal variables, comparing febrile/hypothermic with a confirmed infection source to those in whom no source was found.

### Evaluation of fever and hypothermia

At least one temperature measurement was recorded for 4,176 (99%) of participants, and two or more measurements were recorded for 2917 (69%). Of 205 (5%) febrile or hypothermic participants, 174 (85%) completed physical exam and symptom questionnaire, blood and urine cultures, and malaria RDT. Seven of 205 (3%) withdrew from the study, and seven (3%) refused sample collection and clinical evaluation but did not withdraw. Another 17 underwent partial microbiological evaluation: 11 (5%) were missing urine culture, one (0.5%) was missing blood culture, three (1%) refused sample collection but underwent clinical examination, and two (1%) refused clinical examination and sample collection but completed the symptom questionnaire (Fig 1). The mean number of days between delivery and fever onset was 2.6 (SD 3.8).

### In-hospital postpartum infection incidence

Overall, 84/174 (48%) febrile or hypothermic participants who underwent full clinical and microbiological evaluation met criteria for one or more postpartum infections. Postpartum endometritis was the most common source, identified in 76/193 (39%) who underwent clinical evaluation, including 19 women evaluated clinically but who refused microbiologic evaluation. Of 76 women with clinical endometritis, 61 (82%) delivered by cesarean, 7% of 875 cesarean deliveries. Twenty-five of 175 (14%) participants with urinalysis and urine culture results met criteria for UTI. Bloodstream infection was diagnosed in 5/185 (3%) participants with blood cultures. Another 5/186 (3%) were malaria RDT-positive. Chart diagnosis of cesarean surgical site infection was recorded for 5/205 (2%) febrile or hypothermic participants. The remaining 90/174 (52%) participants did not have a documented source of fever after our evaluation.

### Urinary tract infections

Of 25 UTIs, one (4%) was uncomplicated, five (20%) were non-catheter-associated pyelonephritis, and 19 (76%) were catheter-associated UTIs (CAUTIs), including 5/19 (26%) cases of pyelonephritis and 14/19 (74%) lower-tract UTIs (Table 2). Of all CAUTIs 17/19 (90%) occurred in cesarean-delivery participants. Mean catheter days among CAUTI participants was 1.9 (SD 0.6), which did not differ from non-CAUTI catheterized participants (2.4, SD 1.4, *P* = 0.14). A single pathogen was recovered from 23/25 (92%) UTIs, while two pathogens were recovered in 2/25 (8%). Six (24%) UTIs were caused by Gram-positive organisms, and the remaining 19/25 (76%) by Gram-negatives. *Acinetobacter spp*. were the most common organisms associated with UTIs (11/25, 44%), followed by *Escherichia coli* (5/25, 20%). Nine of 11 (82%) *Enterobacteriaceae* causing UTIs tested positive for extended-spectrum β-lactamase (ESBL) production. Eighteen of 22 Gram-negative rods (82%) were cefepime non-susceptible, and 17/22 (77%) were ciprofloxacin non-susceptible (Table 3). Of six UTIs identified by organisms staining positive on Gram stain, 1/6 (17%) was caused by *Candida* spp., 3/6 (50%) by coagulase-negative *Staphylococcus* spp., and 1/6 each (17%) by *Enterococcus faecalis* and *Enterococcus faecium* (Tables 2 and 4).

**Table 2:** Postpartum infections listed by source of culture-positive sample, including pathogens isolated.

	*URINARY TRACT INFECTIONS* (*N* = 25)		
*Catheter-associated*		*Non-catheter-associated*	
(*n* = 19, 76% of all UTIs^a^)		(*n* = 6, 24% of all UTIs^a^)	
Lower-tract (*N* = 14, 74%)		Lower tract (*N* = 1, 17%)	
**Gram-negative** (*N* = 9)		**Gram-negative** (*n* = 1)	
*Acinetobacter* spp. (*n* = 5)		*Klebsiella pneumonia* and	
*Escherichia coli* (*n* = 3)		*Acinetobacter* spp. (*n* = 1)	
*Klebsiella pneumonia* (*n* = 1)			
**Gram-positive** (*N* = 5)			
Coagulase-negative *Staphylococcus* (*n* = 2)			
*Enterococcus faecalis* (*n = 1)*			
*Enterococcus faecium* (*n* = 1)			
Yeast, probable *Candida* spp. (*n* = 1)			
Pyelonephritis (*N* = 5, 26%)		Pyelonephritis (*N* = 5, 83%)	
**Gram-negative** (*n* = 5)		**Gram-negative** (*N* = 4)	
*Acinetobacter* spp. (*n* = 3)		*Escherichia coli* (*n* = 1)	
*Escherichia coli* (*n* = 1)		*Escherichia coli* and *Acinetobacter* spp. (*n* = 1)	
*Acinetobacter* spp. and		*Klebsiella pneumoniae* (*n* = 1)	
*Pseudomonas aeruginosa (*n *= 1)*		*Bulkholderia cepacia* (*n* = 1)	
		**Gram-positive** (*N* = 1)	
		Coagulase-negative *Staphylococcus* (*n* = 1)	
***BLOODSTREAM INFECTIONS*** (*N* = 5)			
**Gram-negative** (*N* = 4, 80% of all BSIs^b^)		**Gram-positive** (*N* = 1, 20% of all BSIs^b^)	
*Salmonella typhi* (*n* = 1)		*Staphylococcus aureus* (*n* = 1)	
Probable *Veillonella* spp. (*n* = 1)			
*Escherichia coli* (*n* = 1)			
*Acinetobacter junii* (*n* = 1)			

Table 2 footnote

^a^UTIs: urinary tract infections

^b^BSIs: bloodstream infections.

**Table 3:** Antimicrobial resistance in Gram-negative pathogens isolated from postpartum urine and blood culture samples, listed as number and percentage testing non-susceptible (intermediate or resistant) to each antibiotic.

	Ampi-	Ceftriax-		Genta-	Tobra-		Aztre-	TMP/	Cipro-	Erta-	
	cillin	one	Cefepime	mycin	mycin	Amikacin	onam	SMX^a^	floxacin	penem	ESBL^b^
Infection type	(*n*, %)	(*n*, %)	(*n*, %)	(*n*, %)	(*n*, %)	(*n*, %)	(*n*, %)	(*n*, %)	(*n*, %)	(*n*, %)	(*n*, %)
*Genus and species*											
***URINARY TRACT*** (*n* = 22)											
*Acinetobacter* spp. (*n* = 11)	-	-	11 (100)	11 (100)	10 (91)	1 (9)	11 (100)	-	10 (91)	-	-
*Escherichia coli* (*n* = 6)	6 (100)	4 (67)	4 (67)	4 (67)	3 (50)	1 (17)	4 (67)	6 (100)	3 (50)	1 (17)	4 (67)
*Klebsiella pneumoniae* (*n* = 3)	3 (100)	3 (100)	3 (100)	2 (67)	2 (67)	0 (0)	3 (100)	3 (100)	3 (100)	0 (0)	3 (100)
*Pseudomonas aeruginosa* (*n* = 1)	-	-	0 (0)	1 (100)	1 (100)	0 (0)	1 (100)	-	1 (100)	-	-
*Bulkholderia cepacia* (*n* = 1)	-	-	0 (0)	0 (0)	0 (0)	0 (0)	1 (100)	-	0 (0)	-	-
***BLOODSTREAM*** (*n* = 4)											
*Acinetobacter junii* (*n* = 1)	-	-	1 (100)	1 (100)	1 (100)	0 (0)	1 (100)	-	1 (100)	-	-
*Salmonella typhi* (*n* = 1)	1 (100)	0 (0)	0 (0)	0 (0)	0 (0)	0 (0)	0 (0)	1 (100)	0 (0)	0 (0)	-
Probable *Veillonella* spp. (*n* = 1)	-	-	-	-	-	-	-	-	-	-	-
*Escherichia coli* (*n* = 1)	1 (100)	1 (100)	1 (100)	1 (100)	1 (100)	0 (0)	1 (100)	1 (100)	1 (100)	0 (0)	1 (100)

Table 3 footnote:

^a^TMP/SMX: trimethoprim-sulfamethoxazole

^b^ESBL: extended-spectrum β-lactamase.

**Table 4:** Antimicrobial resistance in Gram-positive pathogens isolated from postpartum urine and blood culture samples, listed as number and percentage testing non-susceptible (intermediate or resistant) to each antibiotic.

	Peni-	Ampi-	Chloram-	Cefox-	Clinda-	Erythro-	Genta-	TMP/	Nor-	Tetra-	Vanco-	PCN-ase^b^
	cillin	cillin	phenicol	itin	mycin	mycin	mycin	SMX^a^	floxacin	cycline	mycin	positive
Infection type	(*n*, %)	(*n*, %)	(*n*, %)	(*n*, %)	(*n*, %)	(*n*, %)	(*n*, %)	(*n*, %)	(*n*, %)	(*n*, %)	(*n*, %)	(*n*, %)
*Genus and species*												
***URINARY TRACT*** (*n* = 5)												
Coagulase-negative	3 (100)	-	1 (33)	1 (33)	0 (0)	0 (0)	1 (0)	3 (100)	1 (0)	-	-	2 (66%)
*Staphylococcus* (*n* = 3)												
*Enterococcus faecalis* (*n = 1)*	-	1 (100)	-	-	-	-	1 (100)	-	1 (100)	-	0 (0)	-
*Enterococcus faecium* (*n* = 1)	-	1 (100)	-	-	-	-	1 (100)	-	1 (100)	-	0 (0)	-
***BLOODSTREAM*** (*n* = 1)												
*Staphylococcus aureus* (*n* = 1)	0 (0)	-	0 (0)	0 (0)	0 (0)	0 (0)	0 (0)	0 (0)	0 (0)	-	-	-

Table 4 footnote

^a^TMP/SMX: trimethoprim-sulfamethoxazole

^b^PCN-ase: penicillinase.

### Bloodstream infections

Bloodstream infection was diagnosed in five participants, 4/5 (60%) were Gram-negative (*S*. *typhi*, *Acinetobacter*, probable *Veillonella spp*. and *E*. *coli)*. Two of three (67%) Gram-negative organisms tested for antimicrobial susceptibility were cefepime non-susceptible. One (20%) bloodstream infection was caused by methicillin-sensitive *Staphylococcus aureus* (Tables 2, 3 and 4). No blood cultures were positive for mycobacteria.

### Concomitant infections

Of 174 participants with a complete evaluation, 12/174 (7%) met criteria for both postpartum endometritis and UTI, 3/174 (2%) for postpartum endometritis and bloodstream infection, and none for bloodstream infection and UTI. Of five participants with positive malaria rapid diagnostic tests, 1/5 (20%) met criteria for UTI, 1/5 (20%) for bloodstream infection, and 2/5 (40%) for endometritis. Three of five (60%) participants with a chart diagnosis of cesarean surgical site infection also met criteria for postpartum endometritis.

### Antibiotic prescription and in-hospital mortality

Of 875 cesarean delivery participants who underwent chart review, 788 (90%) were prescribed combination intravenous ceftriaxone and metronidazole postpartum, though there was little documentation of antibiotic receipt. Intravenous ceftriaxone and metronidazole was also prescribed for 70/84 (83%) participants meeting criteria for one or more postpartum infections. Another 11/84 (13%) had no antibiotic prescription, and 3/84 (4%) were prescribed alternative antibiotic regimens. Two of 19 (11%) participants with Gram-negative UTIs, 0/5 (0%) with Gram-positive bacterial UTIs and 2/5 (40%) with bloodstream infections were prescribed an antibiotic to which the bacterium was susceptible. Overall, 802/875 (92%) women delivering by cesarean had a chart-documented prescription for β-lactam perisurgical antibiotic prophylaxis, including 63/71 (89%) of participants with postpartum infection. Antibiotic use in the last year was reported by 149/1,748 women (9%) including 6/82 (7%) with confirmed infection. Report of pre-hospital antibiotic receipt did not differ between febrile/hypothermic participants with 6/82 (7%) or without 5/86 (6%) confirmed infection (*P* = 0.69). Three of 25 participants with UTI (12%) reported antibiotic use within the last year, and of these, urine culture grew *Enterococcus faecalis* in one, yeast cells in the second, and the 3^rd^ grew both multi-drug resistant *Klebsiella pneumoniae* and *Acinetobacter* spp. In-hospital cumulative incidence of postpartum infection did not differ by delivery mode (*P =* 0.20) or HIV status (*P =* 0.27). There were two in-hospital maternal deaths, neither of which were due to infection.

## Discussion

Postpartum infection among rural Ugandan women hospitalized for delivery or postpartum care was most commonly associated with antimicrobial-resistant Gram-negative rods. In-hospital incidence of endometritis, UTI, or bloodstream infection was 2%, and one or more infections were found in 48% of febrile or hypothermic participants. Of 25 Gram-negative bacteria associated with UTI or bloodstream infection, 20 (80%) were multidrug-resistant and cefepime non-susceptible. In this population of postpartum women, *Acinetobacter* spp. were isolated more commonly than any other bacterium. Though *Acinetobacter* are not common causes of urinary tract and bloodstream infection in young, healthy populations, mounting evidence demonstrates the emergence of this pathogen as a cause of hospital-associated and occasionally community-acquired infection in resource-limited settings.[5, 16–20] Receipt of 3^rd^ generation cephalosporins (including ceftriaxone) is a known risk factor for *Acinetobacter baumanii* infection or colonization, and 12/12 (100%) of the participants in whom *Acinetobacter* spp. were isolated in urine or blood cultures were prescribed ceftriaxone. *Klebsiella pneumonia* and *Escherichia coli*, more common causes of UTIs in pregnancy and the postpartum period,[21] also demonstrated high-level antibiotic resistance, with 8/10 (80%) resistant to 3^rd^ and 4^th^ generation cephalosporins. Though some Gram-negative bacterial isolates could be considered contaminants or colonizers, in our cohort samples came from participants with clinical infection and urinalysis findings consistent with UTI, suggesting organisms isolated are true pathogens. The high proportion of antimicrobial-resistant bacteria we report here is concerning, considering only 4/29 (14%) participants were prescribed antibiotics to which their bacterial isolate was susceptible.

Though infection incidence was overall low (2%), we report concerning findings of significant antimicrobial resistance among nosocomial UTIs, which are understudied and likely underestimated in resource-limited settings.[5, 22] The puerperium is especially high-risk for UTI due to physiologic and hormonal changes inhibiting lower urinary tract defenses against invading bacteria.[23] Urethral catheterization is common for cesarean deliveries, and incurs a 3–8% daily risk of bacteriuria, markedly enhancing risk of UTI.[14, 24] In our cohort, urine catheter placement was documented in 96% of cesarean deliveries, for an average of 2 (SD 1) days. Though UTI microbiology and antimicrobial resistance in sub-Saharan Africa is not well described,[4, 5, 25] the high proportion of cephalosporin-resistant Gram-negative bacilli isolated here is concerning for a growing epidemic of antibiotic resistance. Restricting use of urinary catheters to patients with clear indications and removing catheters as early as possible [14] may reduce the incidence of UTIs and limit spread of resistant organisms.

Peri-cesarean antibiotic prophylaxis (a single dose of β-lactam antibiotic) prior to cesarean delivery reduces the incidence of postpartum endometritis threefold.[26, 27] Prophylactic antibiotic prescription was near-universal in our cohort, including those diagnosed with postpartum infection, though most microbiologically-confirmed infections were resistant to common prophylactic regimes. Additional treatment-dose intravenous ceftriaxone and metronidazole was also prescribed to 80–90% of all women delivering by cesarean, though antibiotic receipt was not well-documented. This local practice of administering three days’ intravenous antibiotics followed by five days’ oral antibiotics post-cesarean has arisen from low maternity ward nurse-to-patient staffing ratios (between 1:25 and 1:50) leading to concerns of less timely identification of infections. In addition, lack of reliable microbiology testing makes infection recognition, diagnosis, and appropriate antimicrobial treatment impractical or impossible. Peri-cesarean antibiotic prophylaxis guidelines recommend avoiding therapeutic multi-drug antibiotic regimens, limiting prophylaxis to the simplest and shortest regimens possible to minimize selection pressure for antibiotic-resistant organisms.[27] Antibiotic overuse for obstetric conditions and procedures is common in clinical practice worldwide,[27] and in resource-limited settings such as MRRH, clinicians may not always adhere to these guidelines, rationalizing treatment as prevention. Such use of β-lactam antibiotic treatment regimens at MRRH as presumptive treatment may partially explain high levels of antibiotic resistance and unusual organisms associated with infection in our cohort, and is not unique to this setting[28].

Strengths of our study include the prospective cohort design, capturing nearly all women presenting to MRRH for delivery or postpartum care within the study period, and near-complete microbiologic evaluation of febrile and hypothermic women using a reliable microbiology laboratory. One potential weakness is lack of genital tract cultures, which we chose not to collect, as culture results may not reflect the true pathogens causing endometritis.[10] However, our report of high levels of β-lactam antibiotic resistance in UTIs and bloodstream infections may inform endometritis treatment, which can involve similar pathogens. Our study is also limited by lack of post-discharge fever evaluation. Patients delivering vaginally are often discharged earlier than cesarean deliveries, and our results may reflect early-discharge bias with fewer temperature measurements in vaginal delivery patients and shorter follow-up resulting from shorter hospital stays. Some vaginal-delivery patients may have left the hospital prior to enrollment or temperature measurement, inflating the proportion of cesarean deliveries in our sample. In addition, though no in-hospital infection-related deaths occurred, we were unable to follow participants post-discharge to determine infection outcomes, which would be especially important for infections treated with ineffective antibiotic regimens. Lastly, we did not investigate other causes of postpartum fever in our population. We did not clinically confirm cesarean surgical site wound infection; diagnose dehydration, thrombophlebitis, pneumonia, or viral infection due to resource constraints, which may partially explain why no source of fever was found in 52% of febrile or hypothermic women.[21]

Guidelines encourage consideration of local bacterial resistance when prescribing antibiotics for postpartum infection treatment and prophylaxis,[29, 30] and we expect our findings of highly prevalent β-lactam resistance among postpartum Gram-negative rod infections to alter local practice. Improving adherence to antibiotic prophylaxis and treatment guidelines, expanding antimicrobial stewardship programs and infection control, and increasing the availability of microbiology testing to inform appropriate antibiotic use should become high priorities in Uganda and similar resource-limited settings.[31] Further research should address when and where antibiotic-resistant bacteria are acquired, infection outcomes, and local challenges in guideline implementation.

In conclusion, we demonstrate that infection microbiology among febrile and hypothermic Ugandan women hospitalized for delivery or postpartum care is dominated by antibiotic-resistant Gram-negative rods. Increasing availability of microbiology testing to inform appropriate antibiotic use, strengthening antimicrobial stewardship programs, and improving adherence to guidelines should be high priorities.
